# Eco-Friendly Synthesis of Zirconium Dioxide Nanoparticles from *Toddalia asiatica*: Applications in Dye Degradation, Antioxidant and Antibacterial Activity

**DOI:** 10.3390/nano15020084

**Published:** 2025-01-07

**Authors:** Arumugam Kathirvel, Ramalingam Srinivasan, Sathasivam Harini, Natarajan Ranjith, Govindan Suresh Kumar, Kesavan Lalithambigai, Raji Atchudan, Mohamed A. Habila, Ahmed M. Aljuwayid, Hae Keun Yun

**Affiliations:** 1 Department of Chemistry, K. S. Rangasamy College of Arts and Science (Autonomous), Tiruchengode 637215, Tamil Nadu, India; harinisathasivam@gmail.com (S.H.); ranjithchemist.chem@gmail.com (N.R.); 2 Department of Horticulture & Life Science, Yeungnam University, Gyeongsan 38541, Gyeongsangbuk-do, Republic of Korea; sribt27@ynu.ac.kr; 3 Department of Physics, K. S. Rangasamy College of Arts and Science (Autonomous), Tiruchengode 637215, Tamil Nadu, India; gsureshkumar1986@gmail.com; 4 Department of Physics, K. S. R. College of Engineering, Tiruchengode 637215, Tamil Nadu, India; lallykesavan@gmail.com; 5 Department of Chemistry, Saveetha School of Engineering, Saveetha Institute of Medical and Technical Sciences, Chennai 602105, Tamil Nadu, India; atchudanr@yu.ac.kr; 6 School of Chemical Engineering, Yeungnam University, Gyeongsan 38541, Gyeongsangbuk-do, Republic of Korea; 7 Department of Chemistry, College of Science, King Saud University, P.O. Box 2455, Riyadh 11451, Saudi Arabia; mhabila@ksu.edu.sa (M.A.H.); aaldossrai@ksu.edu.sa (A.M.A.)

**Keywords:** zirconium dioxide NPs, antioxidant, antimicrobial, photocatalytic dye degradation

## Abstract

Zirconium dioxide nanoparticles (ZrO_2_ NPs) have gained significant attention due to their excellent bioavailability, low toxicity, and diverse applications in the medical and industrial fields. In this study, ZrO_2_ NPs were synthesized using zirconyl oxychloride and the aqueous leaf extract of *Toddalia asiatica* as a stabilizing agent. Analytical techniques, including various spectroscopy methods and electron microscopy, confirmed the formation of aggregated spherical ZrO_2_ NPs, ranging from 15 to 30 nm in size, with mixed-phase structure composed of tetragonal and monoclinic structures. UV–visible spectroscopy showed a characteristic band at 281 nm with a bandgap energy of 3.7 eV, indicating effective stabilization by the phytochemicals in *T*. *asiatica*. EDX analysis revealed that the NPs contained 37.18 mol.% zirconium (Zr) and 62.82 mol.% oxygen. The ZrO_2_ NPs demonstrated remarkable photocatalytic activity, degrading over 95% of methylene blue dye after 3 h of sunlight exposure. Additionally, the ZrO_2_ NPs exhibited strong antibacterial effects, particularly against Gram-negative bacteria such as *E*. *coli*, and significant antioxidant activity, with low IC_50_ values for hydroxyl radical scavenging. In conclusion, the green synthesis of ZrO_2_ NPs using *T*. *asiatica* leaf extract is an effective, eco-friendly method that produces nanoparticles with remarkable antioxidant, antimicrobial, and photocatalytic properties, highlighting their potential for applications in water treatment, environmental remediation, and biomedicine.

## 1. Introduction

Materials chemistry places significant emphasis on the design of nanomaterials, including techniques to manipulate the shape, phase, size, and surface properties of metal oxide nanoparticles (NPs) [1,2,3]. Researchers are particularly interested in the above aspects because nanosized metal oxide-based materials exhibit direction and morphology-dependent properties that can be tailored through various nanoparticle growth control techniques. These engineered materials hold promise for applications in drug delivery, biosensing, electrochemical sensing, and catalysis [4].

Zirconium dioxide nanoparticles (ZrO_2_ NPs) are recognized for their unique physicochemical and biological properties, making them highly suitable for applications in biosensing, photocatalysis, cancer therapy, antimicrobial treatments, and diagnostics [5]. Oxide-based semiconductors such as ZrO_2_ are ideal for photocatalytic applications due to their light absorption, charge-transfer properties, and stability. With band gaps and dual electronic structures, these materials efficiently generate charge and absorb light, often enhanced by cocatalysts for redox reactions [6]. ZrO_2_’s high redox potential and wide band gap make it an effective photocatalyst for the mineralization and decomposition of pollutants. Its stability further contributes to its effectiveness in photocatalysis [6,7,8].

There are two primary approaches for synthesizing ZrO_2_ NPs: top-down and bottom-up [9,10]. The top-down approach involves breaking down bulk material into smaller crystallites using high mechanical energy sources like ionic sputtering and milling [11,12]. However, this method has several drawbacks, including changes to the physicochemical properties and surface chemistry of the NPs and the production of secondary byproducts, which hinder the formation of nano-sized particles [5]. Conversely, the bottom-up approach assembles NPs from ultra-small particles like atoms or molecules, allowing precise control over fabrication conditions and results in well-defined nanostructures [13]. Techniques in the bottom-up category include microemulsion, precipitation, hydrothermal, sol-gel, sonochemical, green synthesis, and microwave-assisted methods. However, many of these methods often require hazardous chemicals, involve complex processes, and are associated with limited yield and high energy consumption [14]. The green synthesis method involves biomolecules that stabilize the zirconia precursor into ZrO_2_ NPs [15]. Plant-based synthesis is particularly advantageous due to its simplicity, short synthesis time, and absence of toxic compounds, making it less biohazardous [16,17]. Due to the presence of phytochemicals, plant extracts impart additional functional properties to nanoparticles, which can be beneficial for various applications [18,19].

*Toddalia asiatica* Lam., commonly known as orange climber, is a plant with significant pharmacological potential. Various parts of this plant, such as the roots, are used in folklore medicine to treat various ailments like malaria, cough, toothache, stomach ache, diabetes, and influenza [20,21,22,23,24,25]. The bioactive compounds found in *T*. *asiatica*, including flavonoids, alkaloids, lignans, triterpenes, phenolic acids, and coumarins, facilitate the green synthesis of silver NPs with controlled morphology and enhanced biocompatibility [21,22]. Previous studies have demonstrated the antimicrobial, anticancer, antioxidant, antiplasmodial, antiviral, and mosquitocidal properties of *T*. *asiatica* extracts and their silver nanoparticles [21,22,23,24,25,26,27].

Despite its widespread use in folk medicine across tropical and subtropical regions as a rich source of bioactive compounds, *T*. *asiatica* has remained largely unexplored for green nanoparticle synthesis. This study focuses on *T*. *asiatica* due to its unique phytochemical profile, including flindersine (with antimicrobial, antidiabetic, antilipidemic, and antioxidant properties), 5,7-dimethoxy-8-(3′-hydroxy-3′methyl-1′-butene)-coumarin (an antiplasmodial coumarin), and other bioactive compounds [24,25,26]. Its combination of bioactive properties and traditional use in medicine makes it an ideal candidate for nanoparticle synthesis. The phytochemicals present in *T. asiatica* offer advantages such as enhanced stability, biocompatibility, and antimicrobial activity, positioning *T*. *asiatica* as a promising resource for green nanoparticle synthesis [21,22,23]. Moreover, no research has been conducted on using *T*. *asiatica* extract for the production of ZrO_2_ NPs. Thus, this study aims to address this gap by employing a simple and eco-friendly method to synthesize ZrO_2_ nanoparticles using *T*. *asiatica* leaf extract. Furthermore, we explored the antimicrobial and antioxidant potential, as well as the photocatalytic degradation efficacy of the biosynthesized ZrO_2_ nanoparticles against methylene blue dye.

## 2. Materials and Methods

### 2.1. Chemicals and Reagents

All chemicals utilized in this study were of analytical grade, purchased from Loba Chemie (P) Ltd., Mumbai, India, and were used without further purification. Zirconyl oxychloride (ZrOCl_2_·8H_2_O) and methylene blue dye were obtained from S. D. Fine Chemicals, Mumbai, India. Deionized water was used in all experiments. 2,2-diphenyl-1-picrylhydrazyl (DPPH), phosphate buffer, trichloroacetic acid, ferric chloride, hydrogen peroxide, ethylene diamine tetraacetic acid (EDTA), ascorbic acid, potassium ferricyanide, dimethyl sulfoxide (DMSO), butylated hydroxyanisole (BHA), streptomycin, Müller Hinton agar (MHA), and Müller Hinton broth (MHB) were obtained from Himedia Laboratories, Mumbai, India.

### 2.2. Plant Collection and Extract Preparation

Fresh leaves of *T*. *asiatica* were collected from the *Kanjanmalai* Hills (700-900 MSL, latitude 11.623019, longitude 78.055320), Salem District, Tamil Nadu, India. The collected plant material was authenticated by the Botanical Survey of India (Reference number: BSI/SRC/5/23/2016/Tech.166), Coimbatore, Tamil Nadu, India. A voucher specimen (Specimen number: KSRCAS/CHEM/2016/K-07) was kept at the Department of Chemistry, K. S. Rangasamy College of Arts and Science (Autonomous), Tiruchengode, Tamil Nadu, India. The leaves were examined for microbial infections, washed with running tap water to remove dirt, and then shade-dried at room temperature for 14 days. Subsequently, the dried plant material was powdered using a commercial blender. About 10 g of the processed plant material was boiled with 30 mL distilled water (1:3 ratio) for 30 min. The aqueous extract was filtered through filter paper (Whatman no. 1) and stored at 4 °C until it was used for synthesizing ZrO_2_ NPs.

### 2.3. Synthesis of ZrO_2_ NPs

Twenty-five milliliters of the leaf extract was brought to room temperature and mixed dropwise with an equal volume of 0.1 M aqueous zirconyl oxychloride solution while stirring continuously at 1000 rpm using a magnetic stirrer, resulting in pale-yellow colloids. The synthesized NPs were isolated by centrifugation at 10,000 rpm for 5 min, and the collected precipitate was dried at 80 °C for 3 h in a hot air oven, then calcined at 300 °C for 3 h using a muffle furnace.

### 2.4. Characterization of ZrO_2_ NPs

The synthesized ZrO_2_ NPs were subjected to various spectroscopic and microscopy studies to examine their shape, size, and composition. X-ray diffraction (XRD) analysis was performed using a Panalytical Diffractometer with CuKα (λ = 1.5406 Å) radiation (Panalytical, Malvern, UK) to investigate the crystalline nature of the synthesized ZrO_2_ NPs. Fourier-transform infrared (FTIR) spectroscopy was performed using a Perkin Elmer spectrometer (JASCO, 4100LE, Mumbai, India) to analyze functional groups. The absorption spectrum was documented using a UV–visible spectrophotometer (Labindia, UV 3092, Thane, India). Particle size distribution was determined using a particle size analyzer (Nano Plus, Micrometrics, Kunash Instruments Pvt. Ltd., Thane, India). The size and morphology were observed under a High-Resolution Transmission Electron Microscope (FEI Technai G^2^ 20 s-TWIN TEM, Beijing, China) and Scanning Electron Microscope (TESCAN VEGA 3 SBH, Brno-Kohoutovice, Czech Republic). Elemental composition was obtained using Energy X-ray Spectroscopy (TESCAN VEGA 3 SBH, Brno-Kohoutovice, Czech Republic) attached to SEM.

### 2.5. Photocatalytic Dye Degradation Study

The photocatalytic dye degradation efficiency of the synthesized ZrO_2_ NPs was evaluated using methylene blue dye. Various doses of ZrO_2_ (10, 20, and 30 mg) were mixed with 100 mL of methylene blue dye aqueous solution (50 mg/L) and stirred for 30 min to reach equilibrium. The reaction mixture was exposed to sunlight, and the photocatalytic potential was recorded from sunrise to sunset. Samples (2–3 mL) were collected at 30 min intervals for 3 h, and the optical density (OD) was measured at 662 nm using a UV–visible spectrophotometer (Labindia, UV 3092, Thane, India). The controls for the experiment consisted of two setups: an aqueous solution of methylene blue dye exposed to sunlight (control-1) and a methylene blue dye solution with ZrO_2_ NPs (30 mg) kept in the dark (control-2) [28].

### 2.6. Antibacterial Activity Test

The antibacterial efficacy of the synthesized ZrO_2_ NPs was assessed against three Gram-positive bacteria (*Bacillus subtilis*, *Staphylococcus epidermidis*, and *Staphylococcus aureus*) and three Gram-negative bacteria (*Klebsiella pneumonia*, *Proteus vulgaris*, and *Escherichia coli*) using the agar disc diffusion method [19,29]. Bacterial strains were inoculated in 5 mL of MHB and maintained in a shaking incubator at 120 rpm for 16 h at 37 °C to obtain a seed culture of 1.5 × 10^8^ CFU. Paper discs (5 mm) were loaded with various doses (20, 40, and 60 µg/mL in DMSO) of ZrO_2_ NPs. The bacterial suspension (50 µL) was spread on MHA plates employing a sterilized cotton swab, and ZrO_2_ NP discs were placed at equal distances. DMSO (25 µL/disc) and streptomycin (1 mg/disc) were used as negative and positive controls, respectively. These inoculated plates were incubated at 37 °C for 24 h, and the diameter of the growth inhibition zone (in mm) was measured and recorded.

### 2.7. Antioxidant Activity Test

The antioxidant potential of ZrO_2_ NPs was evaluated against various free radicals, including DPPH radical, hydrogen peroxide radical, hydroxyl radical, and ferric reducing power, following the methods of Srinivasan et al. [29], Bandeira et al. [30], Sujatha et al. [31], and Kathirvel and Sujatha [32], respectively. Different doses of ZrO_2_ NPs (20, 40, 60, 80, and 100 μg/mL) were tested, with BHA and ascorbic acid serving as controls in all analyses.

### 2.8. Statistical Analysis

The experiments were performed in triplicate, and the data are presented as mean ± standard deviation (SD). Statistical analyses, including one-way analysis of variance (ANOVA) and Duncan’s multiple range comparison tests, were conducted using SPSS software (Version 23, IBM, New York, NY, USA) with a confidence level of 95%.

## 3. Results and Discussion

### 3.1. XRD Pattern Analysis

**Figure 1:**
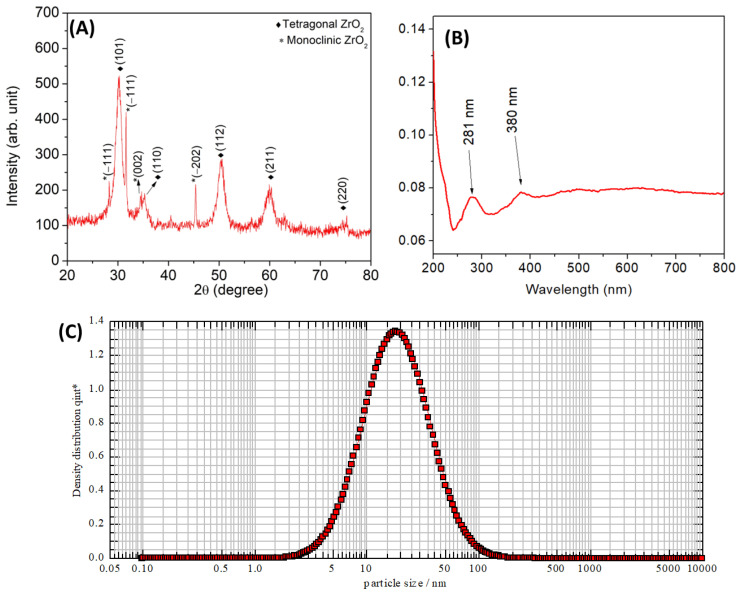
(**A**) XRD pattern, (**B**) UV–visible absorption spectrum, and (**C**) Particle size distribution analysis of ZrO_2_ NPs synthesized using *Todallia asiatica* leaf extract.

The crystalline structure of the synthesized ZrO_2_ NPs was analyzed using XRD patterns recorded in the 2θ range of 20–80° (Figure 1A). The XRD pattern confirmed the successful synthesis of ZrO_2_ nanoparticles with a mixed-phase structure mainly composed of a tetragonal phase showing peaks (101), (110), (112), (211), and (220) with minor contributions of monoclinic phase mineralization represented by peaks (−111), (111), and (−202). These diffraction peaks correlated very much with JCPDS Card Nos. 37-1484 (tetragonal ZrO_2_) and 42-1164 (monoclinic ZrO_2_), confirming the structural identity of the synthesized material [33]. The absence of other peaks indicated the purity of the synthesized ZrO_2_ NPs. The size of the ZrO_2_ NPs was calculated using Scherrer’s formula:

D = 0.94*λ*/*β*cos *θ*

where *λ* is the wavelength of the CuKα radiation (1.5406 Å), *θ* is Bragg’s diffraction angle, and *β* is the full width at half maximum (FWHM) in radians. The size of the ZrO_2_ NPs was calculated using Scherrer’s formula, revealing an approximate crystallite size of 13 nm. Previous studies have demonstrated that plant extracts can produce various phases of crystalline ZrO_2_ NPs, which is consistent with the findings of the present study [33,34,35,36,37,38,39,40,41]. Heating the as-prepared NPs to around 300 °C is necessary to achieve a crystalline form. However, this process may eliminate bioactive molecules from the extracts [10].

### 3.2. UV–Visible Absorption Spectrum

The UV–visible absorption spectrum of ZrO_2_ nanoparticles (Figure 1B) exhibits distinct characteristics of wide-bandgap materials. A prominent absorption peak at 281 nm is observed, which corresponds to electronic transitions, likely from the valence band to the conduction band or oxygen-to-metal charge transfer processes. Additionally, a shoulder around 380 nm suggests the presence of defect-related transitions, such as oxygen vacancies or surface defects [35,36,37]. Beyond this region, the absorption decreases, consistent with the wide-bandgap nature of ZrO_2_. Additionally, the band gap was determined from the UV–Vis absorption spectrum by constructing a Tauc plot, which involves plotting (αhν)^2^ versus hν. The calculated bandgap energy is 3.7 eV which also confirms the formation of ZrO_2_ NPs and these findings are consistent with previous publications [36,37,38,39].

### 3.3. Particle Size Analysis

Particle size analysis revealed that the size of ZrO_2_ NPs ranged from 2 nm to 100 nm. Figure 1C shows the intensity of the particle size distributions of the synthesized ZrO_2_ NPs. This result indicates that formed ZrO_2_ NPs are uniform in shape, with particle sizes found in the range of X_10_ = 7.53 nm, X_50_ = 18.12 nm, and X_90_ = 43.60 nm. The present outcome is in good agreement with previous results where plant extract was used as a stabilizing agent in the synthesis of ZrO_2_ NPs [33,34,35,36,37,38,39].

### 3.4. FTIR Analysis

**Figure 2:**
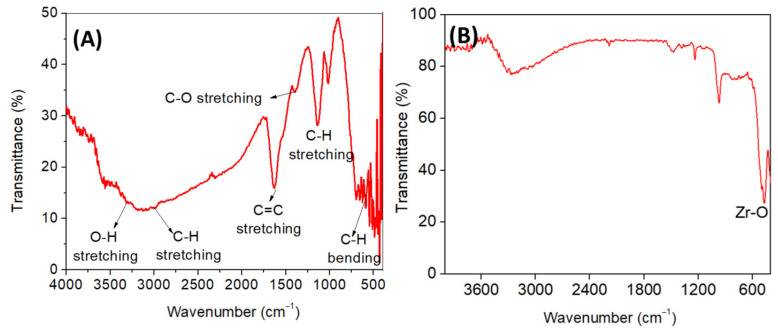
(**A**) FTIR spectrum of *Todallia asiatica* leaf extract and (**B**) synthesized ZrO_2_ NPs.

The FTIR spectra of the *T*. *asiatica* extract and ZrO_2_ NPs are shown in Figure 2A,B, respectively. FTIR spectrum of *T*. *asiatica* extract reveals a broad peak around 3400 cm⁻^1^, indicating O-H stretching from hydroxyl groups (phenolics or alcohols), a peak at 2900 cm⁻^1^, corresponding to aliphatic C-H stretching (terpenoids), a sharp peak at 1627 cm⁻^1^, for aromatic C=C stretching (phenolics or flavonoids), and peaks between 1400–1000 cm⁻^1^, indicating C-O stretching and C-H stretching (carbohydrates, phenols, or esters), along with peaks in the 800–500 cm⁻^1^ region, for aromatic out-of-plane bending, confirming the presence of bioactive compounds like phenolics, flavonoids, terpenoids, and tannins consistent with its medicinal properties [28,37,38,39]. The FTIR spectrum of ZrO_2_ prepared using *Todallia asiatica* leaf extract shows a broad peak around 3400 cm⁻^1^, indicating O-H stretching from adsorbed water or hydroxyl groups, and a peak near 1600 cm⁻^1^ attributed to C=O or C=C stretching, likely from residual phytochemicals in the extract. A strong band around 450–500 cm⁻^1^ confirms Zr-O stretching, verifying the formation of ZrO_2_. The presence of organic functional groups suggests the extract acts as a capping or stabilizing agent during nanoparticle synthesis [40]. The FTIR results suggest that the formation of ZrO_2_ NPs involves the participation of amines, carboxyl groups, phenolic intermediates, carbohydrates, and proteins present in the *T*. *asiatica* leaf extract during the stabilization process. These findings align with previous reports by Goyal et al. [28], Chelliah et al. [37], Selvam et al. [38], and Shinde et al. [39].

### 3.5. SEM-EDAX Analysis

**Figure 3:**
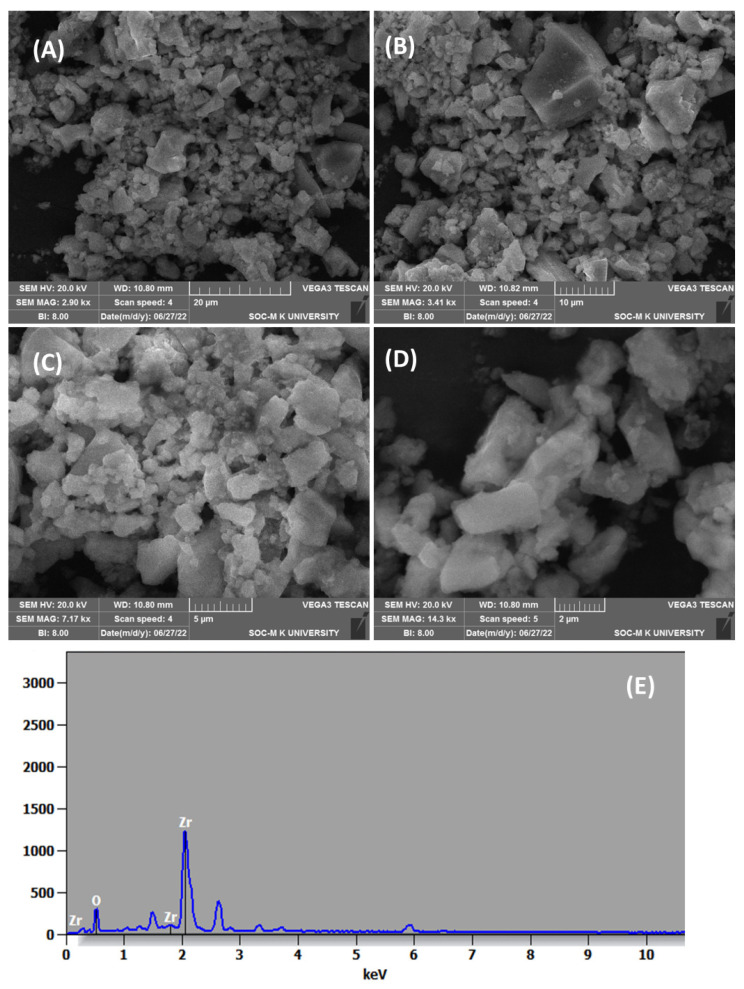
(**A**–**D**) SEM images at different magnifications and (**E**) EDAX spectrum of synthesized ZrO_2_ NPs.

The SEM images shown in Figure 3A–D, confirmed the formation of ZrO_2_ NPs with agglomeration. Agglomerated NPs form through the nucleation and growth of particles on a nanoscale, subsequently interacting with their physical environment [35,36,37]. This small size and tendency to cluster make it challenging to accurately determine the shape and size of individual particles. The EDAX analysis revealed that the ZrO_2_ NPs consisted of 37.18 mol.% zirconium (Zr) and 62.82 mol.% oxygen, indicating nearly stoichiometric composition (Figure 3E). No additional peaks were observed in the EDAX analysis, confirming the high purity of ZrO_2_ NPs. Similar findings with size ranges of ZrO_2_ NPs were observed in previous studies, which strengthen the present results [33,34,35,36]. It is important to consider the presence of oxygen-containing contaminants adsorbed from atmospheric air, which can influence the EDAX analysis of metal oxide materials. To mitigate this, Ar^+^ ion beam sputtering was employed, effectively reducing the adsorbed oxygen concentration to 5–15% in metal oxide materials [41,42].

### 3.6. TEM Analysis

**Figure 4:**
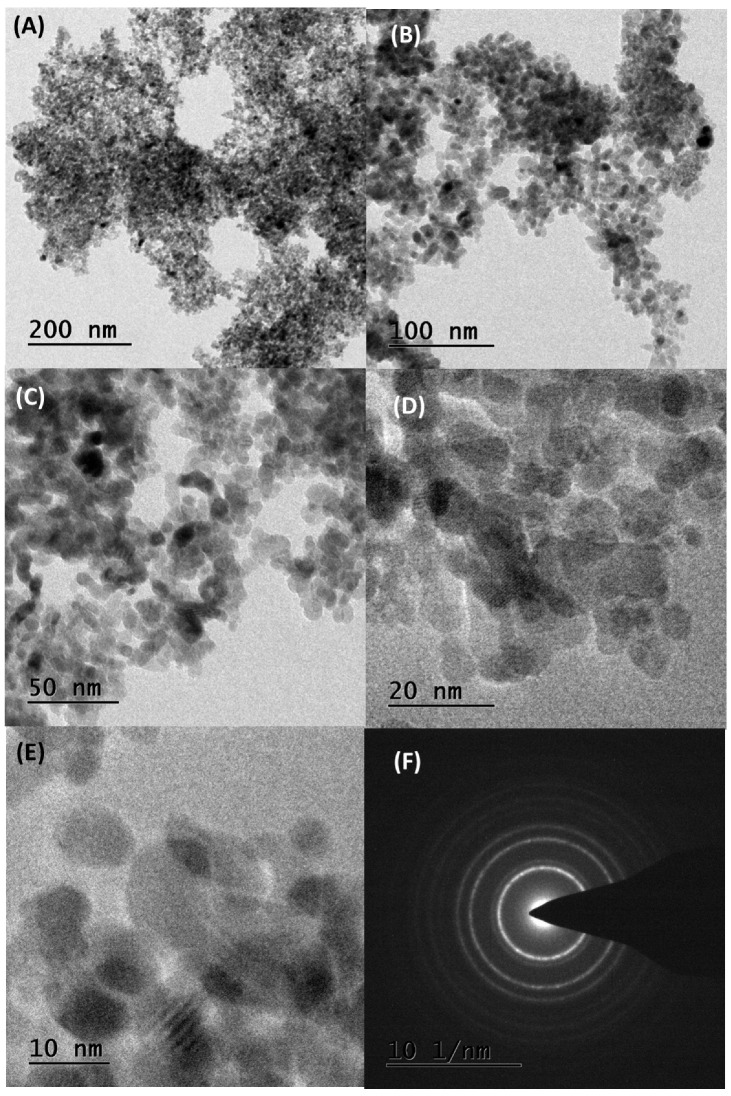
(**A**–**E**) TEM images at different magnifications and (**F**) SAED pattern of synthesized ZrO_2_ NPs.

Figure 4 presents morphological studies conducted via HR-TEM analysis. The majority of observed particles were spherical and exhibited agglomeration. The selected area electron diffraction patterns express distinct spots, indicating sufficient crystallite sizes for effective electron diffraction. The average particle size was estimated to be within the range of 15–30 nm, with a spherical shape. Similar findings have been reported in previous studies, where ZrO_2_ NPs displayed a spherical morphology and mean particle sizes closely correlating with those calculated from XRD patterns using Scherrer’s equation [36,37,38,39]. Literature also indicates that nanomaterials tend to revert to monoclinic Zirconia beyond a critical size range of 20–30 nm [36].

Furthermore, ZrO_2_ NPs ranging from 15–30 nm were found to be uniformly sized and exhibited significant agglomeration, consistent with SEM analysis. The phytochemicals present in *T*. *asiatica* leaf extract were effective in interacting with the Zirconia precursor, crucially controlling the three-dimensional growth of particles to achieve their spherical shape. Previous studies have consistently reported spherical morphology in ZrO_2_ NPs synthesized via green synthesis methods, consistent with our findings [33,34,35]. Notably, the size of ZrO_2_ NPs identified in our study (15–30 nm) is smaller than that reported in other research [43]. Various plant extracts, including *Annona reticulata*, *Averrhoa bilimbi*, *Ficus benghalensis*, *Murraya koenigii*, *Punica granatum*, *Rosmarinus officinalis*, *Sargassum wightii*, and *Wrightia tinctoria*, have been utilized to synthesize ZrO_2_ NPs within the size range of 10–50 nm, aligning with the trends observed in our investigation [37,39,40,41,42,43,44,45,46,47,48].

The overall characterization results suggest that *T*. *asiatica* extract plays a crucial role in determining the size, shape, and crystalline structure of ZrO_2_ NPs through its bioactive phytochemicals [34,35]. Compounds such as flavonoids, phenolic acids, lignans, and alkaloids in the extract likely act as stabilizers, capping agents, and morphology-directing agents, resulting in spherical nanoparticles ranging from 15 to 30 nm in size [34]. Functional groups like -OH and C=O, present in these phytochemicals, may facilitate these interactions, ensuring controlled particle growth and uniformity [34,35].

No additional purification steps were applied to the *T*. *asiatica* extract beyond the standard preparation of aqueous extracts described in the methodology. This approach highlights its eco-friendliness, scalability, and simplicity. The use of aqueous extract instead of organic solvents underscores the strategy of leveraging the diverse bioactive compounds in *T*. *asiatica* as natural stabilizers and capping agents, eliminating the need for hazardous chemicals while reducing cost and time [5,6]. However, these methods may have side effects, including agglomeration, partial oxidation of organic residues, and phase transformations during calcination, as reported in previous studies [5,6,10,28,34,35]. Therefore, the methodology employed in this study aligns well with green chemistry principles, minimizing environmental impact while enhancing the biocompatibility and functionality of the nanoparticles.

### 3.7. Photocatalytic Dye Degradation Activity

**Figure 5:**
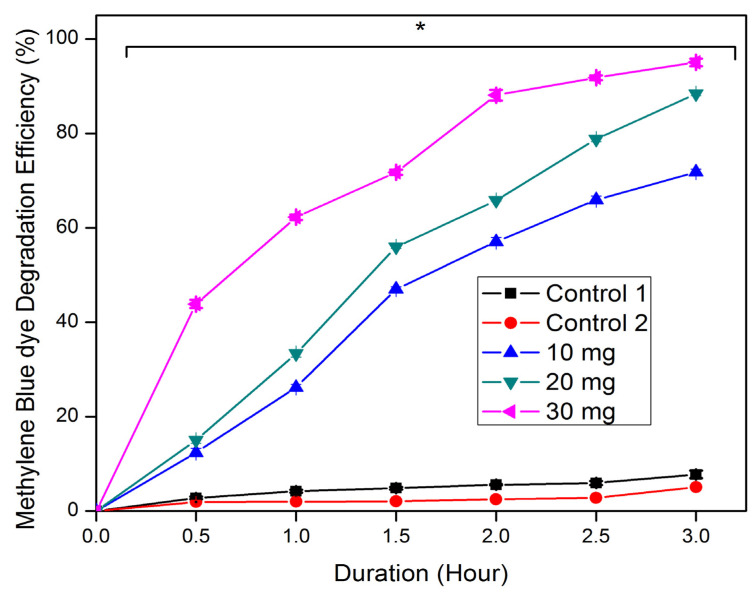
Photocatalytic degradation efficacy of methylene blue dye by synthesized ZrO_2_ NPs at various dosages. Control-1: aqueous solution of methylene blue dye alone exposed to sunlight; Control-2: methylene blue dye solution with ZrO_2_ NPs (30 mg dose) kept in the dark. * Indicates significant differences (*p* < 0.05) based on Duncan’s multiple comparison test.

The photocatalytic activity of ZrO_2_ NPs showed dose- and time-dependent dye degradation potential (Figure 5). A maximum degradation of 95% was achieved with a 30 mg dose of ZrO_2_ NPs over 3 h. Increasing the quantity of ZrO_2_ NPs in the dye solution enhances the reactive sites on the NP surface, thereby augmenting adsorption and facilitating dye degradation [49]. The photocatalytic activity of the synthesized ZrO_2_ NPs is determined by their surface area, with larger surface areas correlating with increased photocatalytic efficiency. In contrast, control-1 without NPs exhibited only 8% dye degradation under light irradiation, highlighting the low electron excitation without ZrO_2_ NPs. Similarly, the control-2 sample (with 30 mg of ZrO_2_ NPs) kept in the dark showed 5% degradation of MB dye. Previous studies on ZrO_2_ NPs synthesized using plant extracts have consistently shown good agreement with our findings regarding their photocatalytic activity for various dye degradation processes [45,46,47,48,49,50,51,52,53]. In this study, the ZrO_2_ NPs demonstrated greater efficiency compared to previous reports, achieving significant degradation within a short reaction time under solar radiation [49,51,54,55,56,57,58].

**Figure 6:**
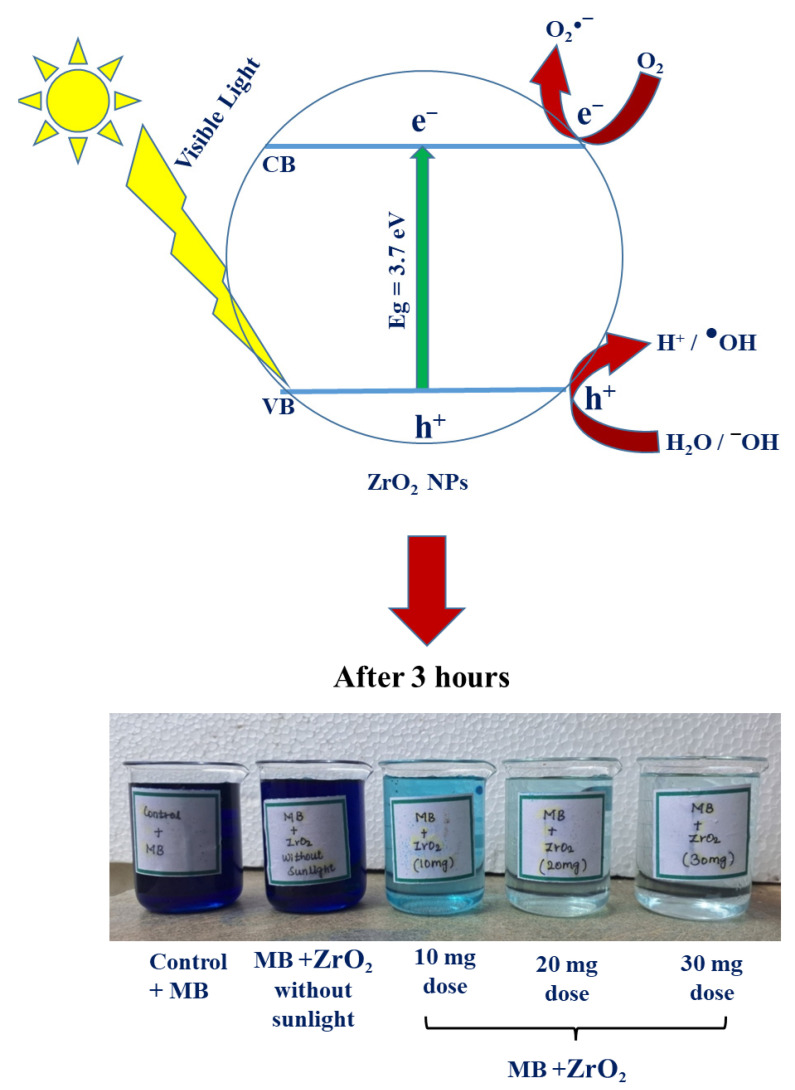
Schematic representation of the photocatalytic degradation of methylene blue dye by synthesized ZrO_2_ NPs exposed to direct sunlight.

Several factors influence the photocatalytic performance of ZrO_2_ NPs, including their size, shape, surface area, antioxidant potential, dopant materials, crystallite size, release of ions, and environmental conditions such as pH, temperature, light intensity, and source [35,36,37,38,39,40]. The photocatalytic mechanism of ZrO_2_ NPs involves excited electrons moving to the conduction band, generating holes in the valence band. These electrons and trapped electrons migrate to the ZrO_2_ NP surface, where they interact with oxygen vacancies. Oxygen vacancies trap O_2_ molecules, producing superoxide radicals (O_2_^●−^). Simultaneously, oxygen from the dye solution also forms superoxide radicals. Meanwhile, holes interact with water or OH^−^ groups, forming hydroxyl radicals (OH^●^). These radicals effectively degrade MB dye [59]. For a visual representation of the photocatalytic dye degradation process using ZrO_2_ NPs, refer to Figure 6. Equations (1)–(5) illustrate the corresponding reactions involving ZrO_2_ NPs, as previously documented by Sindle et al. [39], Chelliah et al. [37], and Modi et al. [60].

(1) $${ZrO}_{2}+Һ\nu \;\to {ZrO}_{2}+e^{-}+h^{+}$$

(2) $$h^{+}+H_{2}O\;\to \;H^{+}+{HO}^{•}$$

(3) $$O_{2}+{2e}^{-}\;\to \;O_{2}^{•-}\;$$

(4) $$Dye+O_{2}^{•-}\to \;Degradation\;Products\;$$

(5) $$Dye+{HO}^{•}\to \;Degradation\;Products\;$$

### 3.8. Antibacterial Activity

**Figure 7:**
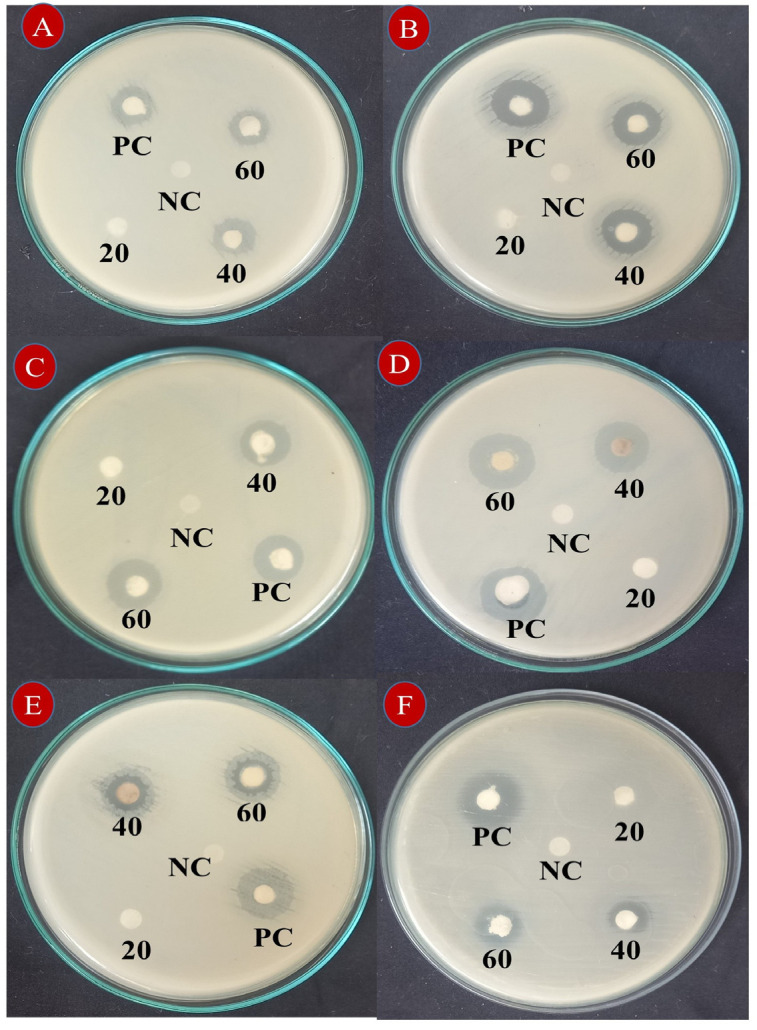
Antibacterial activity of synthesized ZrO_2_ NPs against various bacteria: *Bacillus subtilis* (**A**), *Staphylococcus aureus* (**B**), *Staphylococcus epidermidis* (**C**), *Klebsiella pneumoniae* (**D**), *Proteus vulgaris* (**E**), and *Escherichia coli* (**F**). Numbers (20, 40, and 60) inside the images indicate the doses of ZrO_2_ NPs (in µg/mL); PC = Positive control, streptomycin (1 mg/disc); NC = Negative control, DMSO (25 µL/disc).

The synthesized ZrO_2_ NPs exhibited wide-spectrum, dose-dependent antibacterial activity, significantly inhibiting the growth of both Gram-negative and Gram-positive bacteria (Table 1 and Figure 7). At a high dose (60 µg), ZrO_2_ NPs showed maximum growth inhibition zones of 20.67 ± 0.58 mm for *E*.*coli*, 17.33 ± 0.58 mm for *P*. *vulgaries* and *K*. *pneumoniae*, 14.00 ± 1.00 mm for *S*. *epidermidis*, 12.00 ± 1.00 mm for *B*. *subtilis*, and 12.33 ± 0.58 mm for *S*. *aureus* (Table 1), comparable to the positive control streptomycin.

In contrast, the lowest dose (20 µg) of ZrO_2_ NPs exhibited minimal inhibition zones of bacterial growth, typically ranging from 1–2 mm. The antibacterial efficacy of ZrO_2_ NPs was notably higher against Gram-negative bacteria compared to Gram-positive bacteria, likely due to the absence of a cell wall in Gram-negative bacteria, facilitating easier entry of ZrO_2_ NPs [61,62,63,64]. Several studies have reported the significant antibacterial potential of green-synthesized ZrO_2_ NPs against both Gram-positive and Gram-negative bacteria, supporting our findings [36,64,65,66].

Bacterial cell membranes, primarily composed of negatively charged proteins such as peptidoglycan and other macromolecules [67], interact with positively charged ZrO_2_ NPs through electrostatic interactions [68]. These NPs, characterized by their nanoscale size, spherical shape, high surface area, and biocompatibility, easily penetrate cell membranes [67]. Inside the bacterial cells, ZrO_2_ NPs disrupt metabolic functions, damage proteins, and affect cell membrane permeability, ultimately deactivating the bacteria [47]. This antibacterial mechanism may involve the generation of reactive oxygen species (e.g., O_2_^●−^ and ^●^OH) that cause damage to genetic materials and interfere with cellular processes, leading to bacterial death [69,70].

### 3.9. Antioxidant Activities

**Figure 8:**
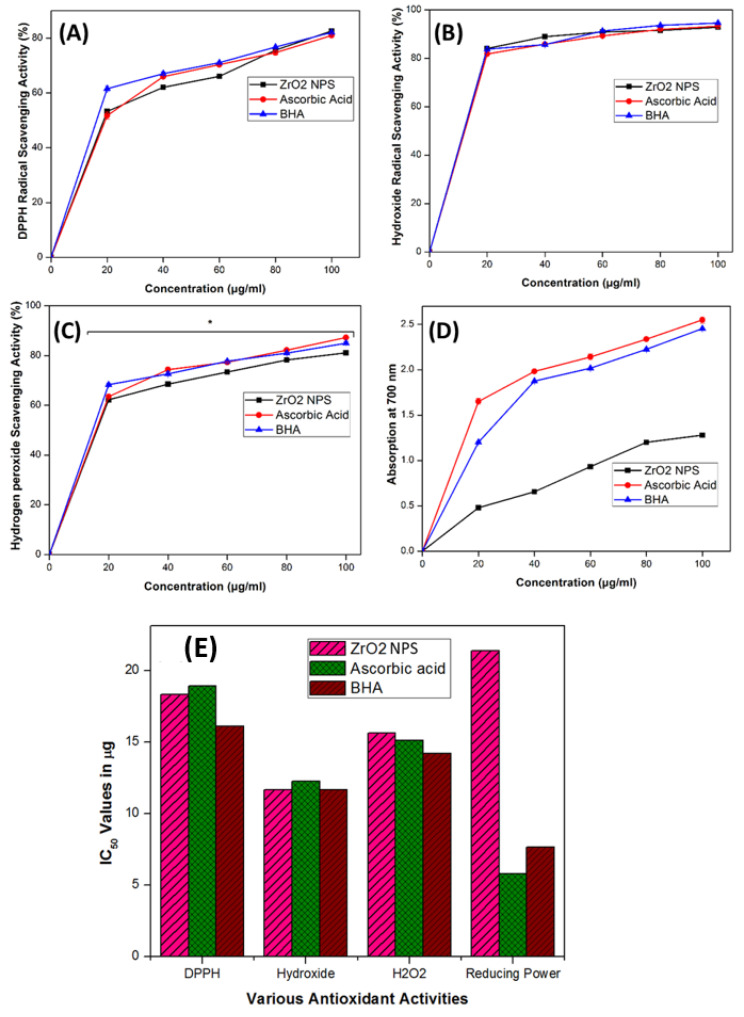
Various radical scavenging potentials of synthesized ZrO_2_ NPs at different doses. (**A**) DPPH, (**B**) hydroxide, (**C**) hydrogen peroxide radical scavenging activity, (**D**) ferric reducing power assay, and (**E**) IC_50_ value comparison. * Indicates significant differences (*p* < 0.05) based on Duncan’s multiple comparison test.

The antioxidant potential of ZrO_2_ NPs plays a crucial role in their photocatalytic activities, demonstrating a positive correlation between these two properties [28,39,71,72]. Antioxidant analysis revealed that synthesized ZrO_2_ NPs exhibited significant antiradical activity, with low half-maximal effective dose (EC_50_) or half-maximal inhibitory dose (IC_50_) values across various assays (DPPH, hydroxyl radical, hydrogen peroxide radical, and FRAP) (Figure 8). The scavenging activity of ZrO_2_ NPs was dose-dependent (Figure 8A–D). Specifically, ZrO_2_ NPs showed superior scavenging potential against hydroxyl radicals compared to other radicals, achieving a maximum IC_50_ value of 11.77 ± 0.15 μg/mL, comparable to ascorbic acid and BHA (Figure 8E). Additionally, ZrO_2_ NPs exhibited sustained antioxidant activity against DPPH and hydrogen peroxide radicals with IC_50_ values of 18.38 ± 0.5 μg/mL and 15.57 ± 0.25 μg/mL, respectively (Figure 8E). However, their ferric reducing capacity showed a higher EC_50_ value of 21.46 ± 0.04 μg/mL, indicating lower reducing potential in this assay. The antioxidant potential of ZrO_2_ NPs synthesized from various biomaterials has demonstrated significant antiradical activity against different radicals, consistent with the present findings [28,60,72].

Previous studies recommend assessing antioxidant capacity using multiple methods for a comprehensive understanding [73]. In our study, we evaluated the antioxidant capacity of ZrO_2_ NPs against four different radicals. Variations in results across DPPH, FRAP, hydrogen peroxide radicals, and hydroxyl radicals may be attributed to differences in sensitivity of ZrO_2_ NPs to each radical species. For example, hydrophilic nature compounds are known to effectively reduce DPPH radicals [74]. This variability underscores the importance of employing diverse antioxidant assays to thoroughly assess the antioxidant potential of materials like ZrO_2_ NPs.

The antioxidant activity of ZrO_2_ NPs is attributed to the electron density of oxygen atoms within the ZrO_2_ NPs, facilitating electron transfer to radicals. ZrO_2_ NPs act as effective electron donors, converting radicals into more stable products, thereby terminating free radical chain reactions and reducing overall radical levels [72,75]. These findings align with the positive correlation observed between the antioxidant, antibacterial, and photocatalytic activities of ZrO_2_ NPs.

## 4. Conclusions

In the present study, ZrO_2_ NPs were successfully synthesized using an eco-friendly method with an aqueous extract of *T*. *asiatica* as a stabilizing agent. The synthesized ZrO_2_ NPs ranged in size from 15–30 nm, were spherical in shape, and exhibited mixed-phase structure composed of tetragonal and monoclinic structure. These NPs demonstrated remarkable photocatalytic potential, resulting in a 98% degradation of methylene blue dye. Additionally, ZrO_2_ NPs showed significant antioxidant and antimicrobial activities against various bacteria and free radicals. The positive correlation between the antioxidant activity of ZrO_2_ NPs and their antibacterial and photocatalytic activities suggests their promising multifunctional capabilities. Future research could explore further applications and mechanisms underlying these beneficial properties, paving the way for their potential use in the biomedical, environmental, and industrial sectors.

## Figures and Tables

**Table 1:** Antibacterial activity of synthesized ZrO_2_ NPs.

ZrO_2_ NPs Dose (µg)	Diameter of Zone of Inhibition (in mm)					
	*B*. *subtilis*	*S*. *aureus*	*S*. *epidermidis*	*K*. *pneumoniae*	*P*. *vulgaries*	*E*. *coli*
20	02.33 ± 0.58 ^b^	00.67 ± 0.58 ^a^	02.67 ± 0.58 ^b^	02.67 ± 0.58 ^b^	01.67 ± 0.58 ^b^	02.67 ± 0.58 ^b^
40	10.33 ± 0.58 ^c^	10.67 ± 0.58 ^b^	09.00 ± 1.00 ^c^	14.67 ± 0.58 ^c^	14.33 ± 0.58 ^c^	10.67 ± 0.58 ^c^
60	12.00 ± 1.00 ^c^	12.33 ± 0.58 ^c,b^	14.00 ± 1.00 ^d^	17.33 ± 0.58 ^d^	17.67 ± 0.58 ^d^	20.67 ± 0.58 ^d^
PC *	17.33 ± 1.15 ^d^	14.67 ± 0.58 ^d^	19.67 ± 0.58 ^e^	18.67 ± 0.58 ^d^	19.67 ± 0.58 ^e^	25.00 ± 1.00 ^e^
NC *	00.00 ± 0.00 ^a^	00.00 ± 0.00 ^a^	00.00 ± 0.00 ^a^	00.00 ± 0.00 ^a^	00.00 ± 0.00 ^a^	00.00 ± 0.00 ^a^

PC * = Positive control, streptomycin (1 mg/disc); NC * = Negative control, DMSO (25 µL/disc). Different superscript letters (a–e) within each column indicate significant differences (*p* < 0.05) based on Duncan’s multiple comparison test.

## Data Availability

The raw data supporting the conclusions of this article will be made available by the authors on request.
